# A DTI-Based Template-Free Cortical Connectome Study of Brain Maturation

**DOI:** 10.1371/journal.pone.0063310

**Published:** 2013-05-13

**Authors:** Olga Tymofiyeva, Christopher P. Hess, Etay Ziv, Patricia N. Lee, Hannah C. Glass, Donna M. Ferriero, A. James Barkovich, Duan Xu

**Affiliations:** 1 Department of Radiology and Biomedical Imaging, University of California San Francisco, San Francisco, California, United States of America; 2 Department of Neurology, University of California San Francisco, San Francisco, California, United States of America; 3 Department of Pediatrics, University of California San Francisco, San Francisco, California, United States of America; Institute of Psychology, Chinese Academy of Sciences, China

## Abstract

Improved understanding of how the human brain is “wired” on a macroscale may now be possible due to the emerging field of MRI connectomics. However, mapping the rapidly developing infant brain networks poses challenges. In this study, we applied an automated template-free “baby connectome” framework using diffusion MRI to non-invasively map the structural brain networks in subjects of different ages, including premature neonates, term-born neonates, six-month-old infants, and adults. We observed increasing brain network integration and decreasing segregation with age in term-born subjects. We also explored how the equal area nodes can be grouped into modules without any prior anatomical information – an important step toward a fully network-driven registration and analysis of brain connectivity.

## Introduction

Characterizing the structure, performance, and plasticity of the human brain network and its trajectory across the lifespan is a fundamental goal of neuroscience. The field of *connectomics* uses non-invasive technologies, including resting-state and task-based fMRI, MEG, and EEG (function), as well as diffusion tractography and morphometric imaging (structure) to map brain networks at the macroscopic scale and thereby allow their analysis using graph theory [1], [2].

While the ultimate goal of connectivity studies is improved understanding of brain function, a strategic way to approach this goal is to characterize the physical connections that mediate information transfer between cortical regions [3]. Refinement of structural brain networks during the course of human development has recently been explored by several groups [4]–[7]. The main observations are summarized in Table 1. Increasing global efficiency and decreasing clustering coefficients (and, hence, local efficiency) were observed in the late developing brain (2–18 years) [4]. Constant global efficiency and increased local efficiency were measured in a longitudinal DTI-based study of subjects at the ages of 2 weeks, 1 year, and 2 years [5]. Another longitudinal study of subjects at 1 month, 1 year, and 2 years, where the brain networks were derived from morphological correlations of brain region volumes, reported increasing global efficiency, and, from 1 to 2 years, increasing local efficiency [6]. Finally, Khundrakpam et al. [7] reported the presence of a critical time window in late childhood (8.5–11.3 years) with increased global efficiency and decreased local efficiency, indicating that structural brain networks may take on a more random configuration during this developmental period, associated with greater plasticity.

**Table 1:** MRI studies of structural brain networks in development.

References	Subjects	Method	Parcellation	Network changes with age
Hagmann et al., 2010 [4]	30 subjects: 2 y–18 y	DTI	66 or 241 nodes,landmark-based	global efficiency ↑, clustering ↓
Yap et al., 2011 [5]	39 subjects: 2 w, 1 y,2 y longit.	DTI	78 nodes, AAL	global efficiency constant, local efficiency ↑
Fan et al., 2011 [6]	28 subjects: 1 mo, 1 y,2 y longit. +27 adults	GM volume correlation	90 nodes, AAL	global efficiency ↑, modularity ↑ from 1 y to 2 y
Khundrakpam et al., 2012 [7]	203 subjects: 5–8 y,8–11 y, 11–15 y, 15–18 y	cortical thickness covariance	78 nodes, AAL	local efficiency ↓, modularity ↓, global efficiency ↑ in late childhood

AAL – Anatomical Automatic Labeling atlas.

The youngest subjects studied were two-week-old term-born neonates [5] (Table 1). Studying brain networks in neonates and infants is a challenging task, as the brain at this time is small, with rapidly changing size, regional topology and myelination. These dynamic features of the developing brain raise the question of how to define the nodes of the brain network or, equivalently, define the cortical *parcellation*. Khundrakpam et al. [7] considered the template-based brain parcellation used in their study to be a methodological limitation, stating that “the use of an alternative with higher resolution which is not constrained by anatomical landmarks is needed in the future.” Fan et al. also noted that anatomical brain regions defined in the atlas used in their study might not match with function and anatomy very well during early brain development [6]. In contradistinction, the recently developed “baby connectome” framework for studying structural connectivity in infants is based on an automated template-free parcellation scheme and can facilitate the mapping of brain networks in the rapidly developing brain [8]. The suggested method utilizes equal area sphere partitioning, a generalized approach that avoids anatomic constraints and is therefore less intrinsically biased than atlas-based approaches. It has been applied in a hypoxic ischemic encephalopathy (HIE) cohort at the age of six months at the University of California San Francisco, in which a correlation between global network properties and neurological outcome was observed [8].

The *purpose* of the present study was to examine the maturational changes of the cortical connectome in subjects across the age spectrum, from premature neonates to term-born neonates, six-month-old infants, and adults using a template-free analysis of white matter connectivity. We hypothesized that the template-free cortical connectome integration and segregation metrics (or global and local efficiency respectively) are different in maturation. Given the partially contradictory results reported by previous studies (Table 1), our hypothesis did not specify whether an increase or decrease in those metrics would be observed with age.

## Results

**Figure 1:**
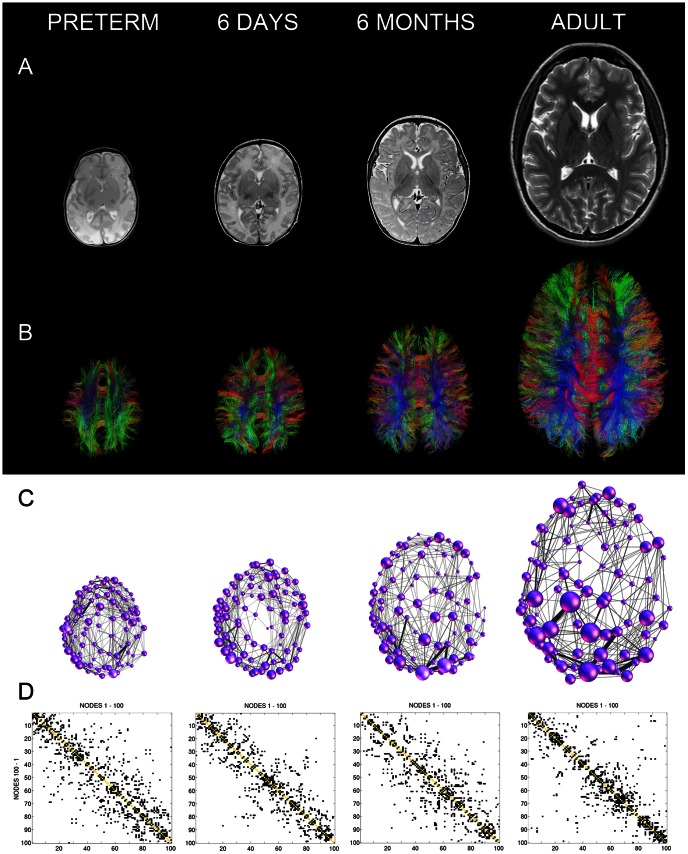
Maturation of the “baby connectome”: examples of brain networks at four different ages. (**A**) Anatomic MRI images (3T, T_2_-weighted fast spin-echo pulse sequence, echo train length 16, TR/TE = 5000/120 ms, 512×512 matrix, in-plane resolution 0.4×0.4 mm^2^, slice thickness 3 mm, 2 averages). (**B**) Tractograms reconstructed based on DTI data. Visualization: minimum length 15 mm, skip 90%. (**C**) Brain networks represented as weighted graphs. The size of the nodes is proportional to the node degree. The edge weights are proportional to the streamline count. (**D**) Binary connectivity matrices, reordered in a way that maximizes the number of connections close to the main diagonal (1,000,000 reordering attempts). *Note: the 6 days and 6 months networks were mapped in the same infant longitudinally.*

**Figure 2:**
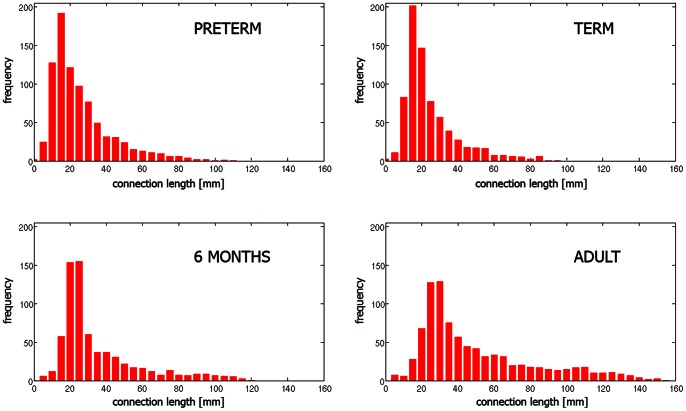
Distribution of physical connection lengths in cortical networks averaged across subjects within each group.

Figure 1 qualitatively illustrates the developmental trajectory of the structural brain network for a representative subject from each group. The following is shown for each subject: an anatomic T_2_-weighted image, a tractogram, a weighted graph, and a reordered connectivity matrix. Differences in the physical size of the brains can be appreciated. The histograms of the connection lengths in cortical networks averaged across subjects within each group (Fig. 2) showed a typical reduction of the frequency over distance [9].

### Changes in Global Topological Properties with Age

**Figure 3:**
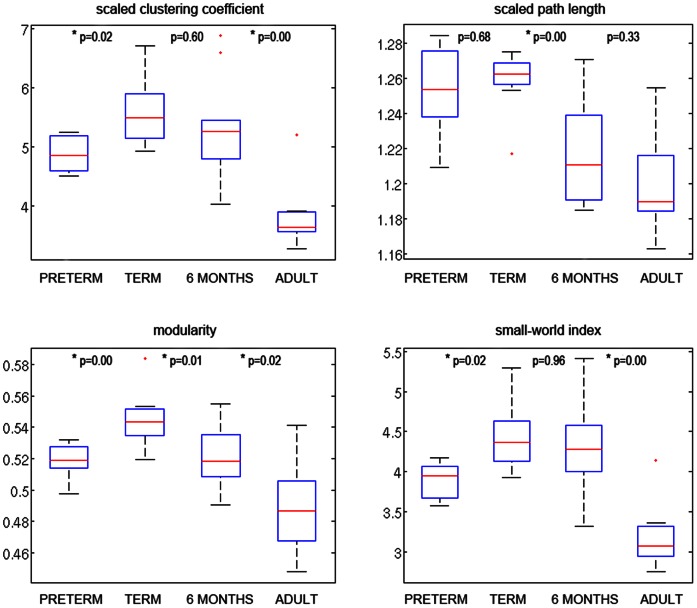
Group analysis of scaled network metrics (scaled clustering coefficient and scaled characteristic path length), modularity, and small-world index. *p<0.05.

**Figure 4:**
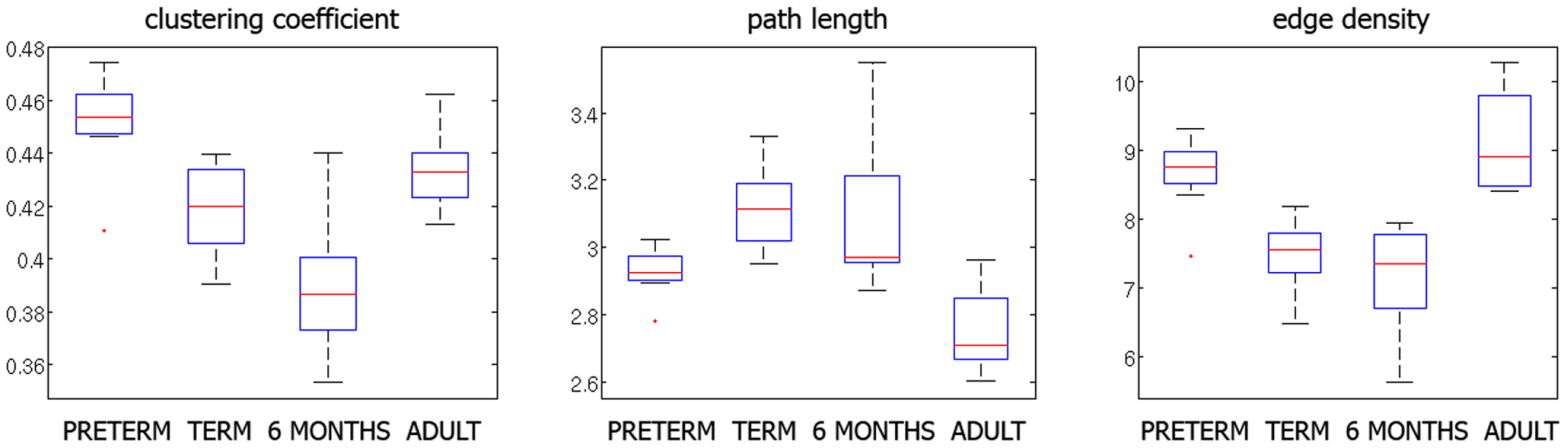
Group analysis of absolute network metrics: clustering coefficient C, characteristic path length L, and edge density. The dependence of C and L on the edge density can be appreciated.

The results of group analysis are shown in Figure 3. The absolute values for the clustering coefficients and characteristic path lengths in the adult group (Fig. 4) were similar to the previously reported values in the adult population based on diffusion tensor imaging (DTI) and atlas-based networks [10]. Since the clustering coefficient and characteristic path length are correlated with edge density, it is crucial that differences between networks’ segregation and integration measures are not just caused by differences in the edge density measure [9]. The edge density, which is the proportion of connections that exists relative to the number of potential connections of a network, varied across groups, ranged from 5.6% to 10.3%, and was higher in the preterm group and adults (Fig. 4). Previously reported values for the cortico-cortical fiber tract connectivity of the mammalian brain ranged from 10% to 30% [9].

We established the integration and segregation metrics by comparing them with null-hypothesis networks that have a random topology but share the size and edge density [11]. The group comparison of the scaled characteristic path length showed a negative trend with increasing age, reflecting increasing integration (Fig. 3). Also, an age-dependent decrease of the scaled clustering coefficient was observed, meaning a decrease in the presence of strongly connected communities, i.e. segregation. Since the decrease in the scaled clustering coefficient was stronger than that of the scaled path length, the small-world index decreased with age (Fig. 3). Our results showed that the clustering coefficients of the structural brain networks were about five times larger than those of random networks, whereas the ratio L/L_rand_ was close to one, leading to a conclusion that networks at all ages displayed small-world properties. Maximized modularity, which similarly to the clustering coefficient reflects the natural segregation within a network, also decreased with age (Fig. 3). Only some of the group differences were statistically significant (Fig. 3).

### Changes in Local Topological Properties with Age

**Figure 5:**
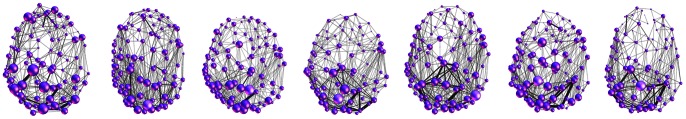
Intersubject variability of graphs within the adult age group. The size of the nodes is proportional to the node degree. The edge weights are proportional to the streamline count.

**Figure 6:**
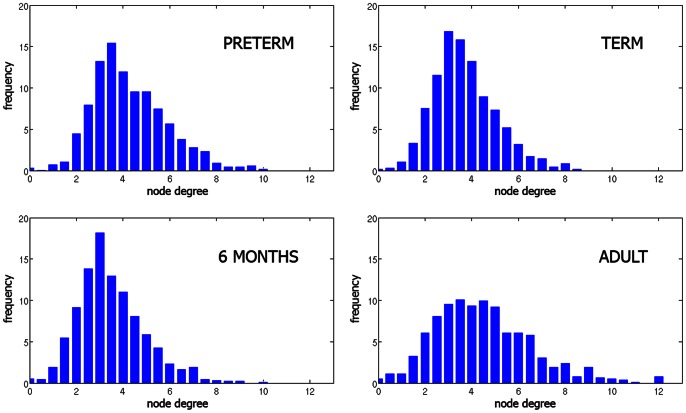
Node degree distribution averaged across subjects within each group.

The spatial distribution of node degree in representative subjects from the four different groups is shown in Figure 1C. The size of the nodes is proportional to the node degree. The intersubject variability of the graphs within the adult age group is shown in Figure 5. Nodes with a large node degree, referred to as network hubs, were located in the posterior cortex in the adults. The node degree distribution in different age groups is shown in Figure 6. Due to the relatively low number of nodes, it was not possible to directly test whether the networks were scale-free [9]. The degree distribution of scale-free networks follows a power law, which indicates a network’s resilience [11]. Nevertheless, we observed highly connected nodes that are unlikely to occur in random networks, this trend being most prominent in the adult group.

### Modules

**Figure 7:**
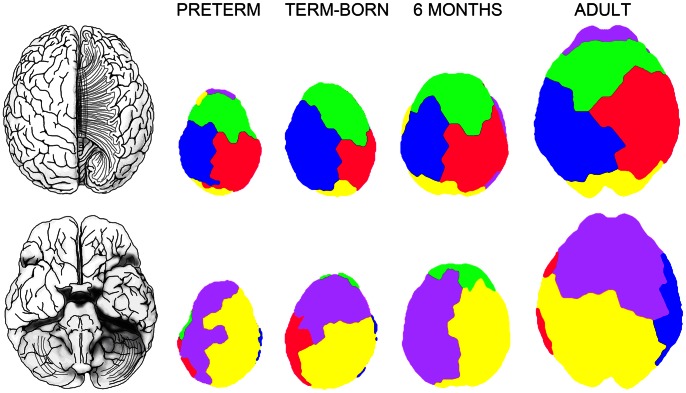
Network-driven segmentation of the cortex into five modules for subjects from all age groups that showed a similar pattern. Top row: dorsal view, bottom row: ventral view. No prior anatomical information was used to find the modules.

Using the maximized modularity, we determined the optimal number of modules that was relatively consistent in all groups: 5–6 modules (average 5.38) in the preterm group, 5–7 modules (average 5.75) in the term-born neonates, 5–8 modules (average 6.6) in the six-month-old infants, and 5–7 modules (average 5.71) in the adults. Under the assumption that the main structural modules are stable across development, we fixed the number of modules to the lower limit of five modules and analyzed the resulting modular structure in all subjects. In Figure 7, we selectively show the obtained network-driven segmentation of the cortex into five modules for subjects from all age groups that showed a similar pattern. It should be emphasized that no prior anatomical information was used to find the modules. The network nodes belonging to the same module were assigned one color and mapped back to the cortex. We observed that spatially adjacent regions tend to belong to the same module [4]. Moreover, correspondence to known anatomy was observed.

## Discussion

In the present study, diffusion tensor MRI was applied to study maturational changes in structural brain connectivity from the first days of life without any assumptions with respect to gyral or sulcal anatomic landmarks. Our main finding is that a basic modular network topology is present in the brain from the first days of life, long before myelination is complete. We also observed increasing brain network integration and decreasing segregation with age in term-born subjects.

One of the principal benefits of using network analysis in neuroimaging research is the provided abstraction that can reduce the complexity and hide features of high variability such as brain size and surface shape to help identify similarities and differences in the organization of neural networks [9]. However, for network representations to be a truly abstract way of looking at neural systems, the following factors have to be eliminated from network models of nodes: the location, size, and functional properties of the nodes. Naturally, anatomical or spatial information can be used in the subsequent analysis, where it can no longer introduce biases. We find this to be especially crucial in studying the developing brain, which motivated our template-free approach. Apart from allowing for abstraction, this approach can lead to finding a network-driven common space for the brain. Finding meaningful anatomical and functional subdivisions of the human brain has been an area of interest since the times of phrenology. The established anatomical parcellations are based on anatomical landmarks or on cytoarchitectonic differences between regions, known as Brodmann’s areas [12]. Interestingly, Brodmann himself noted that the borders of the areas do not match, with a few exceptions, sulci and gyri of the cortical surface or any other external morphological features [13], [14]. By finding brain modules without any prior information and creating an atlas tailored to the individual subject one can account for inter-subject variability within age groups and, importantly for our purpose, study the developing brain. We performed connectivity-based cortical segmentation into modules using a spectral community detection algorithm. Determining the optimal number of clusters is a much-debated problem in the field of community detection. In this study, we used maximized modularity to determine the optimal number of modules that was relatively consistent across groups. Assuming the stability of the main structural modules across development, we used the lower limit of five modules. There was a large degree of variability between subjects, but the resulting parcellations match anatomy reasonably well and can be used as the first iteration in the co-registration of networks (Fig. 7).

The essence of neural function is communication, which is organized around two complementary principles: functional specialization/segregation and functional integration. Network segregation is usually characterized by the average clustering coefficient, whereas network integration is characterized by the characteristic path length. The clustering coefficient reflects the local efficiency of the network (how well neighbors of a node are connected), whereas the characteristic path length reflects global efficiency (how well any two nodes of a network are connected) [9]. Shorter characteristic path length and higher global efficiency have been previously demonstrated to be associated with intelligence [10]. In the present study, the scaled characteristic path length and clustering coefficient decreased with increasing age in term-born subjects (Fig. 3). These results are consistent with a previous study of white matter maturation in subjects between ages two and 18 [4], as well as the most recent study in 439 adolescents and adults [15] and reflect the increasing global brain network efficiency and decreasing local connectivity with age. Also in line with previous observations [4], [15], the small-world index and modularity decreased with age. Prematurely born neonates, however, constituted an exception to these trends. Although the gestational age at scan was below 40 weeks in all cases, many network metrics had characteristics more similar to more “developed” brains than to those of the term neonates imaged in the first days of life. These results can be explained by a non-linear perinatal developmental curve, a different developmental curve in case of prematurely born subjects, or by technical factors associated with imaging this especially challenging group. Comparing network metrics in premature babies with those in term-born babies at the same time point after birth should be investigated in future studies.

By studying the histograms of node degree distribution in different age groups, we observed highly connected nodes that are unlikely to occur in random networks (Fig. 6). This trend was most prominent in the adult group. The spatial distribution of nodes with a high degree (hubs) in adults gravitated toward posterior cortical regions, which is in line with the study of the structural core of the human cortex by Hagmann et al. [16]. More specific localization of the hubs and naming specific anatomic brain regions was not possible due to the nature of the template-free approach. Qualitatively, an asymmetry in hub distribution was observed, with more high-degree nodes being located in the left hemisphere. This tendency has been described previously [16], where, as in our study, all of the adult subjects were right-handed.

### Clinical Applications

Perinatal disease and premature birth affect more than one in 10 of all babies born around the world and put infants at risk of adverse neurodevelopmental outcome [17]. Application of effective therapies in a timely and targeted manner depends on our ability to detect and monitor individual deviations from anticipated normal developmental trajectories. Understanding of the structure, performance, and plasticity of the human brain network and its evolution across the lifespan is a necessary prerequisite to the identification of patients at risk of developmental abnormalities. Some recent studies have utilized whole-brain connectivity analysis in different clinical pediatric populations, correlating global network properties with the outcome [8], [18]. It was shown that intrauterine growth restriction (IUGR) [18] and hypoxic ischemic encephalopathy (HIE) [8] alter brain network topology at one year and six months, respectively, and are associated with the neurodevelopmental outcome. In the long term, our goal is to predict which children are at higher risk of later developmental abnormalities by examining their brain networks shortly after birth, in order to apply appropriate therapies in a timely and targeted manner. In other words, MRI connectomics may become an imaging biomarker of poor neurodevelopmental outcome in infants with prenatal or perinatal diseases. Additionally, monitoring the brain plasticity changes would provide a basis for developing and optimizing therapies to improve outcomes after acquired brain injuries. The present study is crucial in achieving this goal, as it sheds light on the developmental trajectory.

### Methodological Considerations

There are several limitations of the present study that should be pointed out, the first one being the relatively small sample size accompanied by possible differences in etiology within the pediatric groups, all of which were enrolled in the study based on specific clinical conditions (Table 2). In addition, the cross-sectional study design is inferior to a longitudinal observation of brain networks. However, given the rare access to subjects of these ages, we believe that, by characterizing the maturation of template-free brain networks in these subjects, our study provided valuable information contributing to our understanding of how brain structure and function develop.

**Table 2:** Subject and group description.

Group	Subject	Gender	GA at birth	Age at scan
1	P1	F	27 weeks	31.14 weeks PMA
1	P2	M	29.56 weeks	35.14 weeks PMA
1	P3	M	30.28 weeks	33.57 weeks PMA
1	P4	M	30.28 weeks	35.57 weeks PMA
1	P5	F	27.57 weeks	39.71 weeks PMA
1	P6	F	27.14 weeks	31.14 weeks PMA
1	P7	F	29.43 weeks	36.14 weeks PMA
1	P8	F	27 weeks	33.29 weeks PMA
2	BA1	M		6 days
2	BA2	F		4 days
2	BA3	F		4 days
2	BA4	M		5 days
2	BA5	F		4 days
2	BA6	M		14 days
2	BA7	F		5 days
2	BA8	M		1 day
3	BA-6mo1	M		198 days
3	BA-6mo2	M		193 days
3	BA-6mo3	F		211 days
3	BA-6mo4	M		191 days
3	BA-6mo5	F		181 days
3	BA-6mo6	M		202 days
3	BA-6mo7	F		181 days
3	BA-6mo8	F		182 days
3	BA-6mo9	F		185 days
3	BA-6mo10	M		182 days
4	A1	M		30 years
4	A2	F		31 years
4	A3	F		24 years
4	A4	F		24 years
4	A5	F		30 years
4	A6	F		26 years
4	A7	F		30 years

GA – gestational age, PMA – postmenstrual age.

Group 1. Premature neonates (<34 weeks gestational age) excluding: (i) clinical evidence of a congenital malformation or syndrome, (ii) congenital TORCH infection, (iii) newborns too clinically unstable for transport to the MRI scanner.

Groups 2 and 3. Term-born neonates with GA>36 weeks, who had any one of the following: (i) umbilical cord arterial blood pH<7.1, (ii) umbilical cord arterial blood base excess>−10, (iii) Apgar score <5 at 5 minutes of age, (iv) post-asphyxia neonatal encephalopathy syndrome that includes stupor, diminished spontaneous movement, and hypotonia.

Group 4. Healthy adults.

The third group – six-month-old infants – were under anesthesia during the MRI scan. Some neonates were sedated.

Presence of covariables may affect the resulting group analyses. While we used the same diffusion MRI sequence, the b-value varied across the age groups. Typical values of b used in clinical applications range from 600 to 1500 seconds per square millimeter [19]. The lowest values of b are used for imaging of fetuses in utero or premature neonates, due to the very high apparent diffusion coefficient (ADC) values of these structurally immature brains. The traditional adult b-value is 1000 s/mm^2^. In spite of these differences, we expect minimum impact on the resulting scaled network metrics. Another covariable was the brain size. A study by Yan et al. [20] reported that brain size is significantly and negatively correlated with local clustering, suggesting that smaller human brains are more efficient in local information transfer. While their study investigated young healthy adults from a quite narrow age group, our study spans across four very different age groups, whereas the brain size increases across the age spectrum, from premature neonates to term-born neonates, six-month-old infants, and adults (Fig. 1). One may argue that the “development” differences we are describing are just a manifestation of different brain sizes (and, consequently, node sizes) resulting in different network properties. However, our results in premature vs. term-born neonates demonstrate that other effects (presumably, developmental effects) dominate compared to the brain-size related effects.

Another challenging aspect is determining connection strength in weighted networks or setting the threshold in binary networks. In our study, we initially constructed weighted networks with streamline counts used as weights, which for the purpose of network analysis were binarized using a threshold of one streamline. Using binarized networks simplifies the calculation and interpretation of many network measures, but also implies a loss of information of the connectivity pattern. In order to analyze weighted networks, the question of connection weights has to be resolved. Although streamline count is often used as a measure of connection [18], introduction of biases becomes possible since a white matter bundle with higher anisotropy will naturally exhibit a higher count than a bundle with lower anisotropy (FA) [3]. FA itself is another common choice for quantifying the connection strength [10]. Hagmann et al. used the inverse of the average apparent diffusion coefficient (1/ADC) as a multiplicative factor in determining the strengths of interregional pathways [4]. Yet another study by Lo et al. used the product of the streamline count by the mean FA [21]. In the case of probabilistic tractography, the regional connectivity probability can be used as the distance/weight between cortical regions [22]. A recently suggested weighting scheme [23], which scales the number of streamlines by their physical lengths, demonstrated stable intra-class correlation coefficients against thresholding for global efficiency, clustering coefficient and diversity. It can still be argued whether such corrections are sufficient in order to use tractography to provide a quantitative estimate of ‘connection strength’. More comprehensive analysis methods such as *tractometry*, which combines different metrics of white matter microstructure (myelination, axon density, axon diameter) might provide a biologically more meaningful quantification [24].

Thus far, we could directly compare only global network properties, such as the network integration or segregation. The template-free parcellation scheme does not allow for a direct comparison of local properties of single nodes since it does not allow for a straightforward anatomical co-registration of brain networks. However, we are working toward an ambitious goal of finding a network-driven common space for the brain that would allow for fully network-driven inter-subject registration and analysis of brain connectivity. The first step toward achieving this goal was made in this study, in which we explored how the equal area sphere partitioning nodes can be grouped into modules without any prior anatomical information.

Subcortical gray matter structures, such as the thalamus, were not included in the analyzed brain networks, as this would require a precise definition of these structures manually or using templates and, thus, hinder the automated template-free approach to studying the developing brain. Although the influence of these structures on overall connectivity is difficult to define, they might provide valuable information about the course of development. A recent DTI-based thalamocortical connectivity study showed that connections between the thalamus and the frontal cortices, supplementary motor areas, occipital lobe, and temporal gyri are significantly diminished in preterm infants [25]. Therefore, future models should be constructed to include the deep cerebral nuclei.

Finally, since our connectome results rely on the anatomical accuracy of the used tractography methods and their ability to describe white matter trajectories, this sets a fundamental limit on the accuracy of the results. DTI-based connectivity inevitably misrepresents anatomical connectivity to some extent, as it is unable to encode multi-directional diffusion information, resulting in errors in regions where fibers have complex configurations. High angular resolution diffusion models may provide a more accurate white matter tractography result than the simple tensor model by resolving crossing fibers.

## Materials and Methods

### Subjects

All of the MRI scans were compliant with the Health Insurance Portability and Accountability Act (HIPAA) and the study was approved by the Committee on Human Research (CHR) of the University of California, San Francisco. Written informed consent was obtained from all adult participants. In the case of neonates and infants, written informed parental consent was obtained.

The study included three pediatric groups: 8 prematurely born neonates, 8 term-born neonates, and 10 six-month-old infants; the two latter groups included infants with transient encephalopathy at birth, but these had no clinical or imaging evidence of brain injury. The six-month-old infants had a normal neurological outcome assessed at the day of the scan by pediatric neurologists blinded to neonatal course and MR imaging findings. Infants with seizures were excluded from the study. A detailed subject and group description are given in Table 2. Seven healthy adults (age 24–31 years) were included to represent the mature brain.

### MRI Data Acquisition

The subjects were scanned on a 3T GE EXCITE MR scanner using SE EPI with a FOV = 24–25.6 cm, a 128×128 matrix, slice thickness of 1.8–2 mm, 30 directions, and b = 600 s/mm^2^ for preterm babies, b = 700 s/mm^2^ for term and 6-month olds, and b = 1000 s/mm^2^ for adults. Forty-five to 66 contiguous slices were acquired through the entire brain. The scan time for the DTI sequence was approximately four minutes for the babies and nine minutes for the adults. The total time for each examination, which also included T_1_-weighted, T_2_-weighted, and spectroscopic imaging sequences, was approximately one hour. All subjects were scanned in an eight-channel adult head coil. Scan quality with respect to motion and artifacts was visually assessed and subjects excluded accordingly.

### Network Construction

Any network can be represented as a connectivity (or adjacency) matrix, which consists of nodes and edges. The “baby connectome” framework was employed to build connectivity matrices and to assess structural networks [8]. The path from diffusion-weighted images to a connectivity matrix included the following steps.

- A quality assurance step was performed, in which diffusion volumes affected by motion are rejected [8] and remaining images are corrected for eddy current distortions and affine head motion.
- Diffusion tensor reconstruction and deterministic whole-brain streamline fiber tractography was performed using the Diffusion Toolkit software package and Fiber Assignment by Continuous Tracking (FACT) algorithm (threshold angle = 35°).
- Whereas the standard workflow for yielding human connectivity data starts with high-resolution anatomical MRI [9], we based the network construction on extracting the subcortical surface from the non-zero fractional anisotropy (FA) maps. To ensure that tracks would intersect the nodes, the surface 4–6 mm below the cortex was extracted by means of morphological operations (erosion and dilation). It should be noted that the extracted surface included the cerebellum.
- Automated, template-free parcellation of the cortical surface was based on equal area sphere partitioning [26]. The number of equal area nodes was chosen to be 100 based on the network-driven method for determining the optimal number of nodes in six-month-old infants [27]. This method finds the optimum by increasing the number of equal area nodes and for each of the obtained parcellations finding the network’s giant component – the largest connected component [28]. Assuming that all cortical areas of the brain are connected and no part of the brain is structurally isolated, the optimal parcellation – for given population, acquisition, and tractography parameters – is defined as the finest parcellation that still represents the whole brain.
- The output of steps 2 and 4 were combined by computing track-node connections (i.e. when a track touched or intersected the portion of the subcortical surface labeled as a node) and node-node connections and thereby constructing a 100×100 connectivity matrix. Streamline counts were used as edge weights only for visualization purposes. Network visualization was performed using Gephi, an open-source network visualization software package.

Because of noise in the DTI data and oversimplification of the tensor modeling, the fiber tracking may generate many spurious connections that result in false edges in the network [23]. We performed streamline length thresholding on the tractography result prior to constructing the networks. The minimum length of the streamline necessary to be included in the network construction was set to 5 mm for preterm and term-born neonates, as shorter streamlines are likely to be the result of noise. To account for the difference in brain size, this threshold was increased to 10 mm and 15 mm in the 6-month-old infants and adults, respectively [29], [30]. Although 10–15 mm may appear to be a significant length, the following analysis of the physical connection lengths in the obtained networks showed that the effect of this step is negligible (Fig. 2).

### Network Analysis

The resulting weighted networks were binarized (two nodes were considered connected if at least one connecting streamline was present), and network metrics were assessed using the Brain Connectivity Toolbox [11]. Various network metrics were calculated for each network, including edge density, node degree and node degree distribution, maximized modularity (Q), optimal number of modules, average clustering coefficient (C), characteristic path length (L), scaled clustering coefficient relative to a population of random networks (γ = C/C_rand_), scaled characteristic path length relative to a population of random networks (λ = L/L_rand_), and small-world index, defined as a ratio (C/C_rand_)/(L/L_rand_). In the randomized networks, each edge was rewired 1,000 times and an average of 100 networks was used. In addition, the physical connection length of the network edges was calculated as the Euclidian distance between the positions of the two connected nodes, which is a reasonable approximation even for cortical fiber tracks [9]. The connection length distribution was averaged across subjects within each group.

To identify modules of the network, the spectral community detection algorithm was applied [31]. The optimal community structure is a subdivision of the network into non-overlapping groups of nodes in a way that maximizes the number of within-group edges and minimizes the number of between-group edges. The modularity Q is a statistic that quantifies the degree to which the network may be subdivided into such clearly delineated groups. Using Q, we determined the optimal number of modules for each subject. Unlike other network measures, the optimal modular structure for a given network was estimated using an optimization algorithm rather than computed exactly.

Group analysis and regression analysis were performed on the obtained global network properties using Matlab. Due to the exploratory nature of the analyses, the significance threshold was set to 0.05.
